# Gentiopicroside improves high-fat diet-induced NAFLD in association with modulation of host serum metabolome and gut microbiome in mice

**DOI:** 10.3389/fmicb.2023.1145430

**Published:** 2023-08-08

**Authors:** Lijuan Wang, Yuxin Jiang, Qian Yu, Chunping Xiao, Jin Sun, Lili Weng, Ye Qiu

**Affiliations:** School of Pharmacy, Changchun University of Chinese Medicine, Changchun, China

**Keywords:** Gentiopicroside, high-fat diet, non-alcoholic fatty liver, metabolome, gut microbiome

## Abstract

**Objective:**

The incidence of non-alcoholic fatty liver disease is increasing every year, and there is growing evidence that metabolites and intestinal bacteria play a causal role in NAFLD. Gentiopicroside, a major iridoids compound in gentian, has been reported to reduce hepatic lipid accumulation. However to date, no studies have confirmed whether the predominance of Gentiopicroside is related to metabolites and intestinal bacteria. Therefore, we sought to study whether the hypolipidemic effect of Gentiopicroside is related to metabolic function and intestinal flora regulation.

**Methods:**

In the present study, C57BL/6J mice were fed a high-fat diet for 12 weeks, followed by a high-fat diet with or without Gentiopicroside for 8 weeks, respectively. The Gentiopicroside intervention reduced body weight gain, liver index, and decreased serum biochemical parameters such as alanine aminotransferase, aspartate aminotransferase, and triglycerides in high-fat fed mice. The effect of Gentiopicroside on non-alcoholic fatty liver disease was studied using serum untargeted metabolomics and 16S rDNA assay.

**Results:**

Metabolomic analysis showed that the addition of Gentiopicroside significantly altered the levels of amino acids, unmetabolized Gentiopicroside after administration, and metabolites such as Cinnoline, Galabiosylceramide, and Tryptophyl-Tyrosine, which are involved in the pathways regulating bile secretion, tryptophan metabolism, and lipid metabolism. Analysis of intestinal bacteria showed that Gentiopicrosides altered the community composition structure of intestinal bacteria, characterized by an increase and a decrease in beneficial and harmful bacteria, respectively. In addition, correlation analysis showed that the effect of Gentiopicroside on metabolites was positively correlated with intestinal flora *Bacteroides*, *Lactobacillus*, *Muribaculum*, and *Prevotellaceae_UCG_001*. Finally, the combined analysis revealed that metabolites were associated with the regulation of Firmicutes and Actinobacteria and positively correlated with lipid levels.

**Conclusion:**

These results suggest that Gentiopicroside may be a potential agent for the prevention of intestinal disorders and the alleviation of non-alcoholic fatty liver disease.

## 1. Introduction

Non-alcoholic fatty liver disease (NAFLD) is currently the most predominant chronic liver disease worldwide and is a clinicopathological syndrome (Tarantino et al., 2021b; Liou et al., 2022). The prevalence of NAFLD has increased dramatically with the improvement in living standards, and according to epidemiological data, the global prevalence of NAFLD is as high as 25%(Ipsen et al.). Studies have reported multiple pathogeneses of NAFLD, which are associated with hepatic steatosis, insulin resistance, obesity, inflammation, altered innate immune regulation, and the enterohepatic axis, among other factors (Powell et al., 2021). The lipid accumulation and the inflammation caused by NAFLD can be reversed in the early stages of the disease, but without intervention, it may progress to cirrhosis or hepatocellular carcinoma in later stages, both of which pose a serious risk to life and health (Heida et al., 2021). There are currently several clinical trials for NAFLD, but approved drugs are yet to be developed (Matsui et al., 2021). The current reliance on host self-regulation, exercise, and, to a lesser extent, pharmacotherapy for lipid-lowering and anti-inflammatory purposes is unsatisfactory and difficult to maintain in terms of patient compliance (Neuschwander-Tetri, 2020). Therefore, there is an urgent need to identify safe and effective treatments to aid global public health. There is an increasing body of evidence linking intestinal flora disorders to the development of NAFLD pathologenesis (Houghton et al., 2016). Compared to healthy organisms, the structure and composition of intestinal microorganisms are significantly altered in NAFLD patients and mice (Henao-Mejia et al., 2012; De Minicis et al., 2014).

Gentiopicroside (GPS), a type of iridoid glycosides component extracted from the Chinese herbal medicine *Gentiana manshurica* Kitag., *Gentiana scabra* Bunge., *Gentiana triflora* pall or *Gentiana rigescens* Franch., is also the main active ingredient of this herbal medicine and has anti-inflammatory, hypolipidemic, and antioxidant effects (Zhao et al., 2015; Jin et al., 2020; Zou et al., 2021). In addition, GPS has hepatoprotective effects and promotes bile secretion and other biological activities (Chen et al., 2018; Zhang Y. et al., 2021). However, the mechanism by which GPS induces its hepatoprotective effect is unclear, especially given that we have limited knowledge regarding the relationship between metabolites and gut microorganisms in NAFLD.

Recent studies have found a close association between metabolites and intestinal flora and NAFLD pathogenesis (Ji et al., 2019). Moreover, the intestinal flora affects host metabolic phenotypes and is implicated in host co-metabolic processes. This co-metabolic relationship is important for maintaining the physiological health of the host. Importantly, liver pathology occurs when there are disorders of the flora (Bian et al., 2017; Chen and Vitetta, 2020). Recent data indicate that the intestinal microbiota may be a metabolic organ involved in regulating host metabolism. The association between the microbiota and NAFLD pathogenesis has made these small organisms a key focus of NAFLD research (Gart et al., 2016). In addition to changes in the composition of the gut microbiota, components and metabolites from the gut microbiota are key factors in regulating NAFLD pathology (Jia et al., 2021). The gut microbiota produces a variety of bioactive substances that interact with host hepatocytes via the portal vein. These substances include components derived from bacteria, such as lipopolysaccharides, peptidoglycans, DNA, and extracellular vesicles, as well as metabolites ranging from short-chain fatty acids, indole and its derivatives, trimethylamine, secondary bile acids to carotenoids and phenolic compounds (den Besten et al., 2013; Hersoug et al., 2016; Baxter et al., 2019; Wang et al., 2019; O’Riordan et al., 2022). The mechanism by which the liver responds to gut microbiome bioactives is related to the regulation of glycolipid metabolism, immune signaling responses, and redox homeostasis (Boursier et al., 2016). Therefore, the intestinal flora and its metabolites have become important targets for NFALD therapies. Moreover, a comprehensive analysis between the gut microbiome and metabolism in NAFLD mice after GPS treatment may help us to reveal the complexity of NAFLD and provide new therapeutic targets and strategies (Tarantino et al., 2021a).

Therefore, in this study, we performed untargeted metabolomics and 16S gut microbiome sequencing studies on high-fat diet (HFD) -induced mice sera. Herein, we reveal changes in gut microbiota homeostasis as well as serum metabolism in NAFLD mice after GPS supplementation. In addition, we constructed a map of the correlation between the gut microbiota and blood metabolism, revealing possible combinations of key gut microbial metabolites and laying the foundation for further studies on the disease mechanisms of NAFLD.

## 2. Materials and methods

### 2.1. Materials

The GPS standard was purchased from Xi’an Beijinuo Biotech Co., Ltd (Xi’an, China). The HFD was purchased from Research Diets Ltd (No. D12492), USA. Blood alanine aminotransferase (ALT), aspartate aminotransferase (AST), and triglycerides (TG) kits were purchased from Nanjing Jian Cheng Co (Nanjing, China).

### 2.2. Animal treatment

The 40 male C57BL/6J mice, 6 weeks old, with a body mass of 20 ± 5 g, were obtained from Liaoning Changsheng Biotechnology Co (Liaoning, China), License number [SCXK (Liao) 2020-0001]. The feeding temperature was 22 ± 1°C with a humidity of 45%, and the mice had *ad libitum* access to water. All animal experiments strictly complied with the provisions of the Animal Protection Society.

After 1 week of adaptive feeding, the animals were randomly divided into four groups. The control group (Con, *n* = 8); model group (Mod, *n* = 8); low-dose group (Low, *n* = 8); and high-dose group (High, *n* = 8), respectively. The Con group was fed normal food, and the other groups were fed a HFD (D12492, a synthetic food supplemented with 0.15% cholesterol and 60% fat energy) for 12 weeks. The low-dose and high-dose groups were given 400 mg/ (kg * d) and 200 mg/ (kg * d) GPS for 8 weeks, respectively (Xiao et al., 2020). Control mice were given an equal volume of pure water. All animals were weighed every 2 weeks. After 8 weeks, the mice were sacrificed after collecting stool, blood, and liver tissue. Collected samples were immediately stored at −80°C until later use. All protocols regarding diet, anesthesia, blood, and tissue sample collection, and disposal of dead animals were approved by the Experimental Animal Ethics Committee of Changchun University of Traditional Chinese Medicine (No. 2021220).

### 2.3. Biochemical analysis

Blood samples were centrifuged at 3,500 rpm for 10 min at 4°C to separate the serum. Serum TG, ALT, and AST levels were determined on an enzyme-labeled analyzer (Thermo Scientific Co.) using a microplate method.

### 2.4. Histological analysis of liver tissues

Liver tissue samples were fixed in 4% paraformaldehyde, routinely processed, embedded in paraffin, cut into 5 μm sections, and stained with hematoxylin and eosin (HE) for histological analysis. Frozen sections (8 μm) were subjected to pathological analysis using standard procedures for the use of Oil Red O staining kits (Solarbio Life Sciences Co., Beijing, China).

### 2.5. Metabolomic analysis

Three of the eight mice were randomly selected for metabolomic analysis. The mice serum samples were first thawed at 4°C, then vortexed for 1 min before thorough mixing. Then the appropriate amount of sample was transferred into a 2 mL centrifuge tube before addition of 400 μL of extraction solution (methanol acetonitrile volume ratio = 1:1, internal standard concentration 2 mg/L). Samples were then sonicated for 10 min in an ice-water bath and then left for 1 h at −20°C. Following incubation, samples were then centrifuged for 15 min at 4°C, 12,000 rpm. The supernatant was transferred to a new centrifuge tube, concentrated, and dried under vacuum. Next, 160 μL of the extract (acetonitrile-water volume ratio: 1:1) was added before samples were vortexed for 30 s, sonicated for 10 min in an ice-water bath, and centrifuged at 4°C for 15 min at 12,000 rpm. Finally, 120 μL of the supernatant was carefully removed from the 2 mL injection bottle and 10 μL of each sample was mixed into QC samples for testing on the machine.

The liquid chromatography system for metabolomics analysis consisted of a Waters UPLC Acquity I Class PLUS ultra-performance liquid chromatography tandem with a Waters UPLC Xevo G2-XS QTOF high-resolution mass spectrometer, using a Waters Acquity UPLC HSS T3 column (1.8 μm 2.1*100 mm). The mobile phase A: 0.1% formic acid aqueous solution; mobile phase B: 0.1% formic acid. Acetonitrile was used as the detection solvent. The ion source was an ESI source with the following parameters: the ion source temperature was 100°C, the dissolvent gas temperature was 500°C, and the dissolvent gas flow rate was 800 L/h. The backblast gas flow rate was 50 L/h, the cone hole voltage was 30 V, the capillary voltage was 2,500 V, and the scan range (m/z) was 50-1,200.

The raw data, collected using MassLynx V4.2, were processed by Progenesis QI software for peak extraction, peak alignment, and other data processing operations, and metabolites were identified based on the online METLIN database of Progenesis QI software. Fold change analysis, cluster analysis, and KEGG pathway enrichment analysis, and graphing were performed using R-3.1.1 software, GraphPad Prism 9.0 software. Student’s *t*-test was used for statistical significance (*p*-value). Metabolites with a final fold change ≥ 1, VIP value ≥ 1, and *p*-value < 0.05 in the project were considered differentially expressed metabolites. The MetaboAnalyst 5.0^1^ database was used for pathway analysis.

### 2.6. Gut microbiota analysis

Three of the eight mice were randomly selected for intestinal flora analysis. To analyze the intestinal microbiota, fresh fecal samples from the mice were collected and processed according to standard procedures. DNA was extracted using the PowerSoil^®^ DNA Isolation kit, and PCR amplification of the bacterial 16S full-length region was performed using the upstream primer; AGRGTTTGATYNTGGCTCAG, and downstream primer; TASGGHTACCTTGTTASGACTT. The marker genes were sequenced using single molecule real-time sequencing (SMRT Cell) based on the PacBio sequencing platform, followed by filtering of Circular Consensus Sequencing (CCS) sequences. Lima v1.7.0 software, cut adapt 1.9.1 software and UCHIME v4.2 software were used for data pre-processing. Species annotation and abundance analysis were also performed, which can reveal species composition within the samples. Finally, α-diversity, β-diversity, significant species difference analysis, and correlation analysis were performed to uncover the differences between samples.

### 2.7. Correlation analysis and statistical analysis

Statistical analysis and graphing were performed by R-v3.6.1 software, SPSS software version 21.0, and Origin 2022 software for correlation plot analysis, inertia analysis, differential metabolite-differential microbial string plots, and differential metabolite-differential microbial network plots. Pearson correlation coefficients were used to assess the correlation between genus-level gut microbiota and serum metabolites or fecal metabolites. One-way analysis of variance (ANOVA) and two-tailed Student’s *t*-test were applied to determine statistically significant differences. All data are expressed as the mean ± standard deviation. *P*-values < 0.05 were considered statistically significant.

## 3. Results

### 3.1. GPS reduced HFD-induced lipid accumulation in mice

**FIGURE 1:**
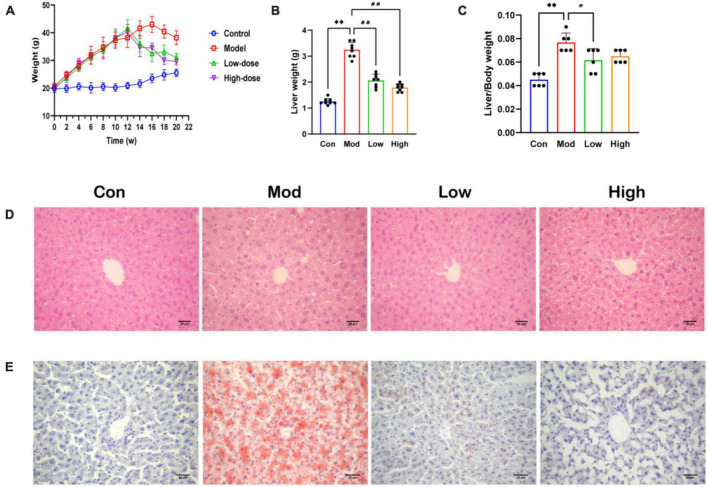
Effect of GPS on fat accumulation and body weight in HFD-fed mice. **(A)** Body weight **(B)** Liver weight **(C)** Index of liver **(D)** HE staining diagram **(E)** Oil Red O staining diagram. Data are presented as the mean ± SD (*n* = 8). ^**^P < 0.01 compared to the Con group; ^#^*P* < 0.05 and ^##^*P* < 0.01 compared to the Mod group.

Body weight increased significantly in the Mod group compared to the Con group (Supplementary Table 1). Importantly, GPS treatment reduced this weight increase, especially in the GPS high-dose group in which mice maintained a similar body weight to animals in the Con group (Figure 1A). The same trend was observed for the liver index and for the ratio to body weight (Figures 1B, C). Administration of GPS after a HFD significantly improved liver performance. HE and Oil Red O staining showed increased hepatocyte balloon degeneration as well as increased red-stained lipids in the HFD group. However, in the HFD+GPS group, lipid accumulation in liver tissue was reduced (Figures 1D, E). Thus, GPS ameliorated the hepatic lipid accumulation and weight gain caused by HFD.

### 3.2. Supplementation with GPS improved blood lipids and liver function

**FIGURE 2:**
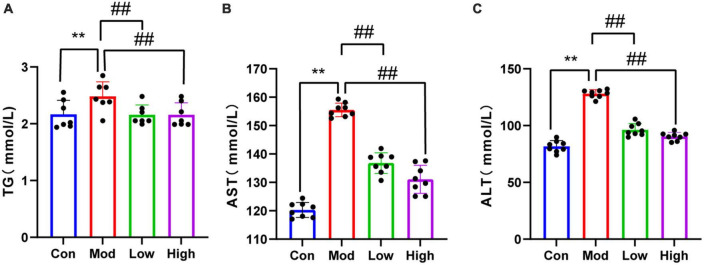
Lipid results of all four groups of mice. Compared to the Con group, HFD-fed mice showed elevated **(A)** TG, **(B)** AST, and **(C)** ALT levels, indicating that the HFD group had abnormal lipid metabolism and liver injury. Supplementation with GPS reduced liver function impairment. **P < 0.01 compared to the Con group; ^##^*P* < 0.01 compared to the Mod group.

Serum TG, ALT, and AST levels were significantly higher in the HFD group compared to the Con group (*p* < 0.01). Notably, GPS administration significantly reduced serum levels of TG, ALT, and AST in mice fed on a HFD after all three doses (*P* < 0.01) (Figure 2).

### 3.3. GPS treatment alters the metabolite composition of HFD-fed mice

**FIGURE 3:**
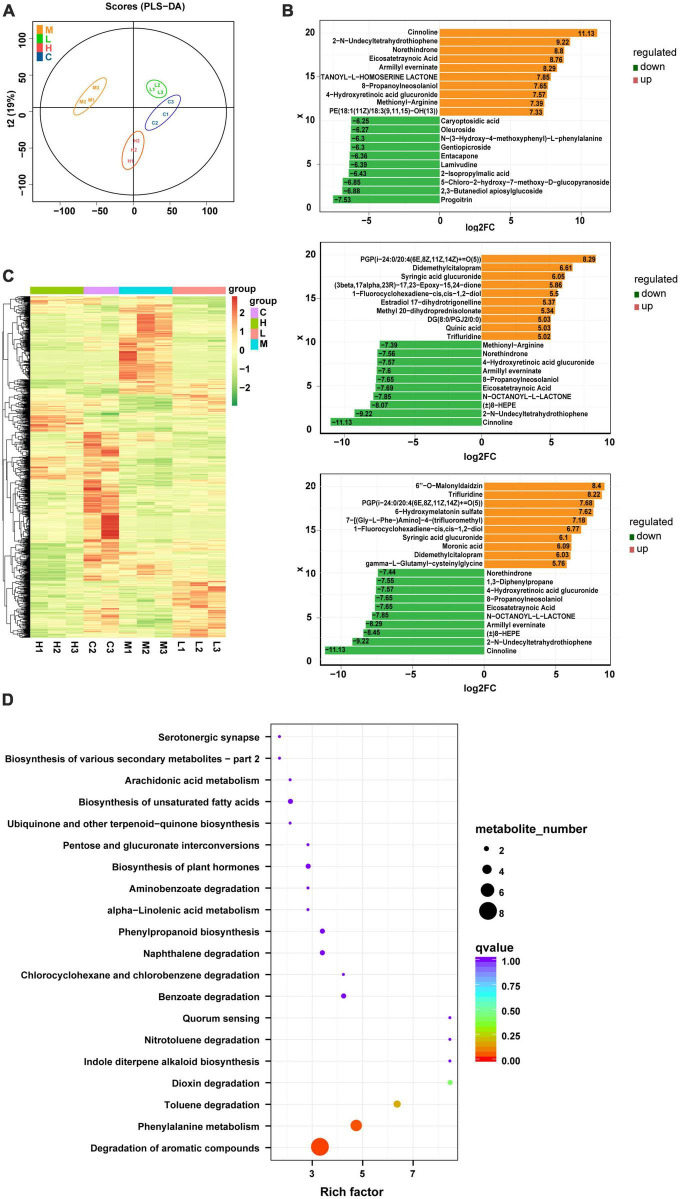
Metabolite composition of all four groups of mice. **(A)** OPLS-DA analysis, **(B)** top 10 metabolite logFC, **(C)** volcano plot, and **(D)** enrichment of metabolic pathways.

Using the LC-Q-TOF platform, qualitative and quantitative metabolomic analyses were performed on 11 samples with metabolites detected in positive ion mode. A total of 7,542 peaks were detected, of which 2,469 metabolites were annotated. The OPLS-DA analysis (Figure 3A) shows a good separation of samples between groups, indicating a significant difference between the four groups. After qualitative and quantitative analyses of the detected metabolites, the top 10 metabolites with logFC values adjusted upward and downward can be compared by first comparing the differences in the fold change (FC) in the quantitative metabolite data for each grouping (i.e., Con & Mod, Mod & Low, and Mod & High comparisons). We found that compared to the Con group, the top 10 upregulated metabolites in the serum of the Mod group were Glabrin D, 5(S)-Hydroperoxyeicosatetraenoic acid, 2-Butyl-3-phenyl-2-propen-1-al, 1,3-Diphenylpropane, Androsta-4,16-dien-3 -one, 5-hydroperoxy-15-HETE, methionyl-alanine Methionyl-Alanine, Oleoyl Ethyl Amide, Oenanthic ether, and dCDP (Figure 3B). Furthermore, the top 10 down regulated metabolites were Eicosapentaenoic acid, 1,4-Benzothiazine-O-quinonimine, Irilone de Iris, Hovenidulcioside B1, 2-Methyl-3′-hydroxyphenylpropionic acid, Propionylcholine, cis-4- Hydroxycyclohexylacetic acid, N-Phenyl-1-naphthylamine, Coumarin, and 5,7,4′-Trihydroxy-3′-methoxyflavanone 4′-O-isobutyrate metabolites (Figure 3B). In addition, cluster analysis revealed that the differential metabolites were upregulated in all treatment groups, with metabolites such as sugars, glycosides, and amino acids predominating (Figure 3C). Finally, a total of 331 metabolites were identified between the Con and Mod groups based on the joint screening of VIP values, logFC values, and *P*-values, with 130 upregulated and 201 downregulated metabolites. These differentially expressed metabolites could be enriched in pathways such as degradation of aromatic compounds, biosynthesis of unsaturated fatty acids, pyrimidine metabolism, bile secretion, and glycerophospholipid metabolism, as detailed in Figure 3D. There are clearly many differentially expressed metabolites between the Mod and Con/GPS groups. In addition, we found significant differences in some metabolites between the four groups. Specifically, on the one hand, the levels of differential metabolites were significantly higher in the Mod group compared to the Con group and could be significantly reduced by GPS supplementation. On the other hand, when mice in the Mod group were fed a HFD diet, the levels of differential metabolites were significantly lower. However, supplementation with GPS reversed their abnormal levels, which may explain the protective effect of GPS.

### 3.4. GPS treatment alters the gut microbiome composition of HFD-fed mice

**FIGURE 4:**
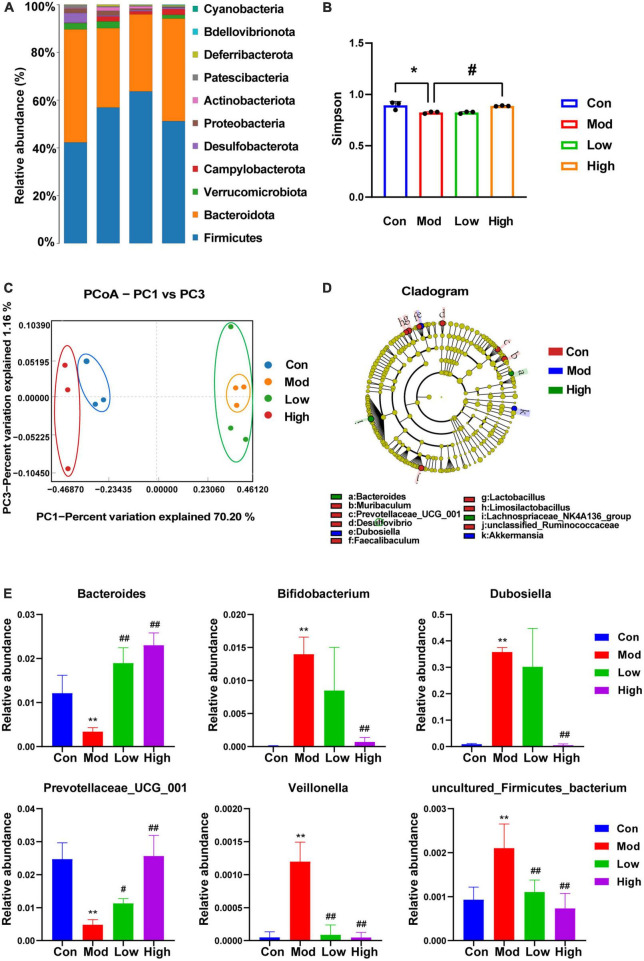
16s rDNA sequencing showing altered microbiota composition after GPS treatment. **(A)** Species distribution histogram, **(B)** Simpson, **(C)** PCoA, **(D)** LEfSe at the genus level, **(E)** ANOVA with variance. *P < 0.05 and **P < 0.01 compared to the Con group; ^#^*P* < 0.05 and ^##^*P* < 0.01 compared to the Mod group.

Microbial diversity was analyzed on the PacBio sequencing platform, using the SMRT Cell method to sequence marker genes, followed by filtering, clustering or denoising of CCS sequences, and species annotation and abundance analysis, which can reveal the species composition of samples. The species composition of the samples can be revealed by filtering, clustering or denoising the CCS sequences and performing species annotation and abundance analysis. Furthermore, α-diversity, β-diversity, and significant species difference analyses were performed. As shown in Figure 4A, compared to the Con group, the main phyla of bacterial species distribution in the Mod and GPS administration groups at the genus level included the thick-walled phylum Firmicutes, the bacteriophage Bacteroidota, Verrucomicrobiota, Campylobacterota, Desulfobacterota, Proteobacteria, Actinobacteriota, Patescibacteria, Deferribacterota, Bdellovibrionota, and Cyanobacteria, among others. Among them, the phylum Firmicutes is the largest bacterial group in the body and many members of this phylum are beneficial bacteria. The phylum Actinobacteriota is associated with diet and with high fat and high protein intake (Jandhyala et al., 2015). Moreover, most species within this phylum are antagonistic. Actinobacteria have been associated with various diseases such as obesity and diabetes with reduced numbers of bifidobacteria in all stages of life (Hidalgo-Cantabrana et al., 2017). Bifidobacteria help to improve digestive problems, improve glycemic control, lower lipid levels, improve immunity, and exhibit antioxidant activity. Our data suggest that a HFD induces changes in the above-mentioned bacteriophage homeostasis and that GPS treatment association with modulates these changes. Analysis of α-diversity (Figure 4B) revealed a significant decrease in Simpson values after 12 weeks of HFD induction in mice, as well as a significant increase in the high-dose administration group. This indicates that the diversity of intestinal microorganisms was upregulated in the high-dose administration group. β-diversity analysis can be achieved through primary coordinate analysis (PCoA), which can reflect the similarities and differences between the display groups. Increased similarity in species composition is illustrated by data points being close together in the coordinate system. The opposite is also true for samples with lower the similarity. From the PCoA analysis plot (Figure 4C), it can be seen that the Con group, Mod group, and GPS Low and High groups are clustered separately. In addition, the Con group is significantly separated from the Mod group, indicating significant changes in the intestinal flora of the mice in the Mod group. Moreover, the Mod group is further away from the Con group, while the High group is closer to the Con group. Therefore, the intestinal flora of the Mod group of NAFLD changed significantly, and the intestinal flora composition of mice was closer to that of the Con group after GPS treatment. Therefore, GPS has the effect of regulating the composition of intestinal microbiota in NAFLD mice. Genus-level based LEfSe analysis (Figure 4D) revealed a significant decrease in the abundance of *Acetobacteroides, Adlercreutzia, Akkermansia, Alistipes, Allobaculum*, and *Bifidobacterium* and an increase in the genera of Gram-negative phragmo bacteria Bacteroides and Acidobacteria in the Mod group compared with the Con group; the opposite was true for the administered group compared with the model group, suggesting that the treatment of hepatobiliary injury was achieved by regulating the abundance of the above-mentioned bacterial groups. After ANOVA analysis (Figure 4E), *Bacteroides, Bifidobacterium, Dubosiella, Prevotellaceae_UCG_001, Veillonella*, and *uncultured_Firmicutes_bacterium* were found to be the main reasons for the differential expression of the four groups of samples.

### 3.5. Correlation analysis between metabolites and intestinal flora

**FIGURE 5:**
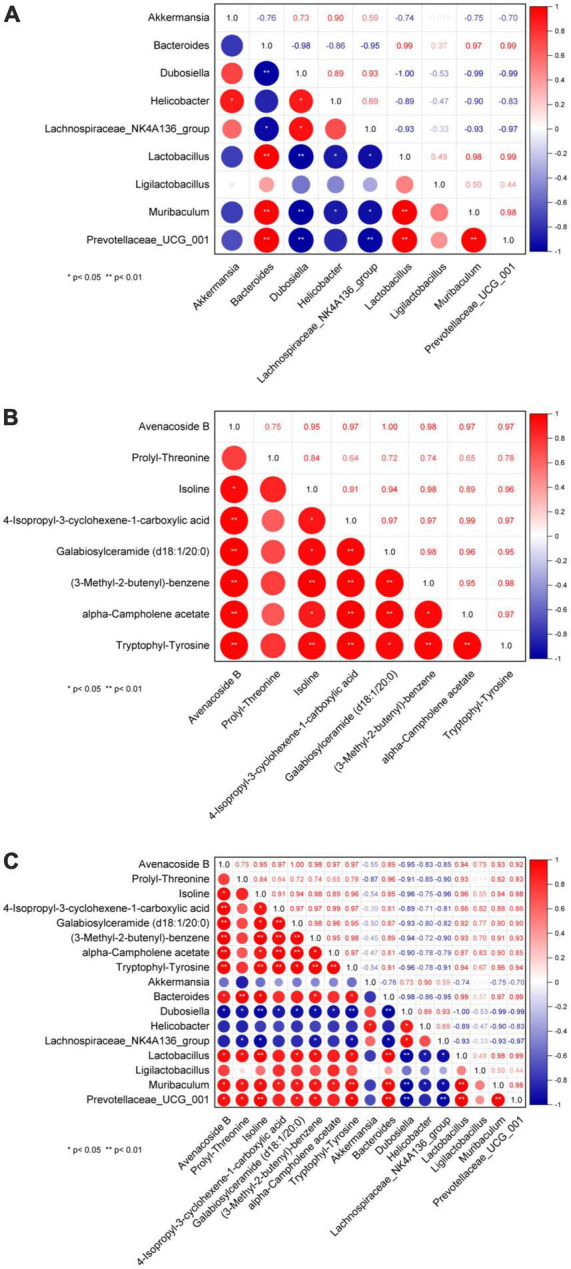
Plot of correlation between genus level intestinal flora and serum metabolites. **(A)** Correlation between flora, **(B)** correlation between metabolites, **(C)** correlation between flora and metabolites. *P < 0.05 significant correlation, **P < 0.01 highly significant correlation.

One hypothesis states that metabolites are upstream factors of intestinal flora. If true, this would confirm that disruption of the intestinal flora in NAFLD can be improved by metabolite regulation. Therefore, to investigate the metabolic mechanism(s) of GPS improvement in NAFLD, we sought to further understand the correlation between metabolomic products and intestinal flora using serum and fecal metabolomics and intestinal flora analysis (Figure 5). A significant correlation was identified between *Bacteroides, Lactobacillus, Muribaculum*, and *Prevotellaceae_UCG_001* in the intestinal flora (Figure 5A). Furthermore, Avenacoside B was significantly correlated with 4-Isopropyl-3-cyclohexene-1-carboxylic acid, Galabiosylceramide (d18:1/20:0), (3-Methyl-2-butenyl)- benzene, alpha-Campholene acetate, and Tryptophyl-Tyrosine (Figure 5B). We also identified a correlation between Avenacoside B, Prolyl-Threonine, Isoline, Galabiosylceramide (d18:1/20:0), (3-Methyl-2-butenyl)-benzene, and Tryptophyl-Tyrosine in the intestinal flora (Figure 5C). Bacteroides, Dubosiella, Lactobacillus, Muribaculum, and Prevotellaceae_UCG_001 were significantly correlated with each other. Among them, Dubosiella was the only negative correlation, all others were positive.

### 3.6. Interaction of metabolites with intestinal flora

**FIGURE 6:**
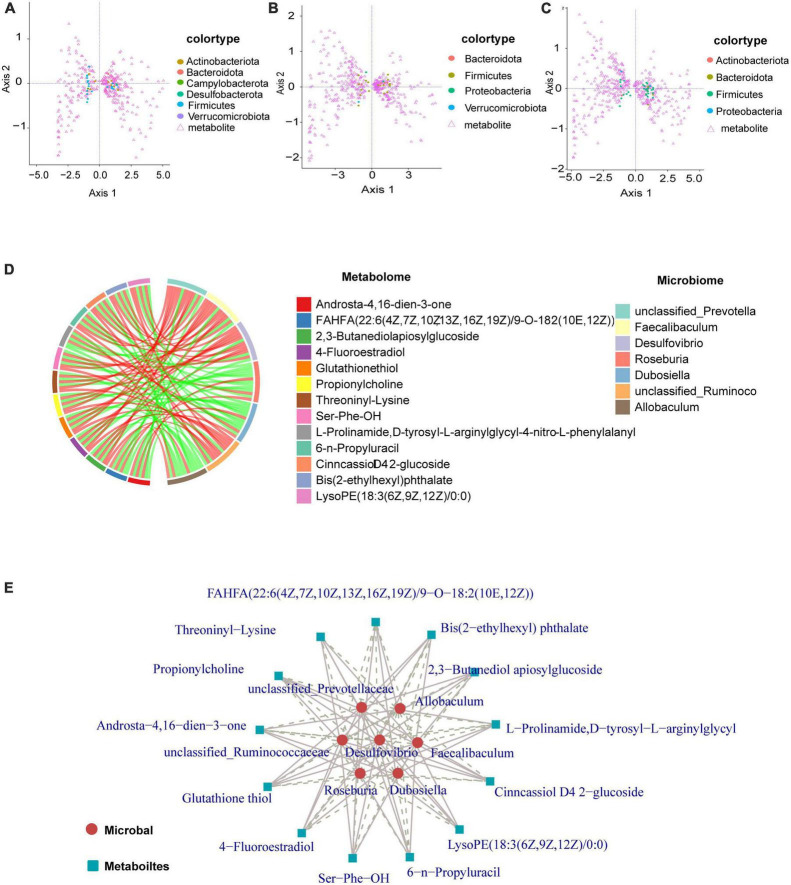
Graphical representation of the interaction of metabolites with intestinal flora. **(A–C)** Circles represent a microorganism (genus level), different colors indicate different classifications (divided by phylum, sorted by phylum name, the first 15 phyla show the corresponding phylum name, other phyla are commonly referred to as *other). Triangles indicate a metabolite. The angle formed by the line connecting the microorganism, metabolite, and the origin reflects the correlation between the metabolite and microorganism. An acute angle is a positive correlation and an obtuse angle is a negative correlation. A right angle is indicative of no correlation. **(D)** There are two forms of chord diagrams: chord diagrams with file names containing labels, where the names of differential metabolites/microorganisms are shown outside the circles. Chord diagrams with file names containing a legend, where differential metabolites and differential microorganisms are shown on the right. The left half of the circle of the chord diagram is the differential metabolite, and the right half of the circle is the differential microorganism. Each chord indicates that the differential metabolite is significantly correlated with the differential microorganism, with red chords representing positive correlations and green chords representing negative correlations. **(E)** Metabolites are marked with blue squares and microorganisms are marked with red circles. Solid lines represent positive correlations and dashed lines represent negative correlations.

Coinertia analysis (Figure 6) can be used for the analysis of two sets of variables and can be applied to the joint multi-omics analysis. Selecting differential microorganisms (genus level) and classifying microorganisms according to phylum, combined with differential metabolites can reveal the relationship between differential metabolites and microorganisms (genus level), and the distribution of microorganisms among different taxa. Generally, the distribution of microorganisms in similar groups is relatively concentrated, and the distribution of similar microorganisms in different differential groups may be different. Our data show that the distribution of microbial taxa between the control and model groups was more dispersed, and mainly Firmicutes and Actinobacteriota were associated with the regulation of metabolites (Figure 6A). From the results in Figures 6B, C, the distribution of microbial taxa between the model group and the low and high dose groups was more concentrated compared to Figure 6A, indicating that the microbial taxa were altered after GPS supplementation, with Firmicutes bacteria being mainly responsible for metabolite regulation. The differential metabolite-differential microbial chord plot is shown in Figure 6B. A table of correlation results for the top 30 frequencies of differential metabolites/differential flora, retaining data containing at least one set of correlation coefficients with absolute values within the top 30 (ordered by the absolute value of correlation coefficients from largest to smallest), was used for the correlation chord diagram (Supplementary Table 2). The differential metabolites identified included Androsta-4,16-dien-3-one, FAHFA(22:6(4Z,7Z,10Z13Z,16Z,19Z)/9-O-182(10E,12Z)), 2,3-Butanediolapiosylglucoside, 4- Fluoroestradiol, Glutathione thiol, Propionylcholine, Threoninyl-Lysine, Ser-Phe-OH, L-Prolina- mide, D-tyrosyl-L-arginylglycyl-4-nitro-L phenylalanyl, 6-*n*-Pro- pyluracil,CinncassioD42-glucoside, Bis(2-Ethylhexyl)phthalate, and LysoPE(18:3(6Z,9Z12Z)/0:0) with the gut microbiome remaining unclassified. The following microorganisms were also found to have a significant correlation: *Prevotella, Faecalibaculum, Desulfovibrio, Roseburia, Dubosiella, unclassified_Ruminoco*, and *Allobaculum*. The differential metabolite-differential microbial network for these is shown in Figure 6C. To further clarify the correlation between metabolism and microorganism, the correlation coefficient table of differential metabolite-differential microorganism and the chord diagram were used for the correlation network diagram. As a result, we can more clearly understand the close association, as well as the positive and negative correlations of *unclassified Prevotella, Allobaculum, unclassified_Ruminoco, Desulfovibrio, Faecalibaculum, Roseburia*, and *Dubosiella* with the differential metabolites (Figure 6E).

## 4. Discussion

Non-alcoholic fatty liver disease is a multifactorial disease with a complex pathogenesis including liver alterations with increased lipid accumulation, with or without inflammation, and without other causes of liver disease (Pafili and Roden, 2021). NAFLD is fast becoming an emerging global health challenge. Exercise and dietary interventions are the only available strategies for NAFLD treatment (Gao et al., 2020) as there are no approved first-line drugs for prevention or treatment (Roeb, 2021). GPS is a cyclic enol ether terpenoid with many biological activities. Cyclic enol ether terpenoids have been shown to exist mainly as prototypes and glycosides as well as I and II metabolites. Importantly, these metabolites have been shown to have hepatoprotective, anti-inflammatory, antitumor, hypoglycemic, and hypolipidemic activities (Wang et al., 2020). Moreover, recent studies have suggested that GPS may also have significant ameliorative effects on NAFLD through hypolipidemic and anti-inflammatory effects (Jin et al., 2020). In the present study, we performed LC-ESI-MS/MS-based metabolomics combined with 16S rRNA gene sequencing. To our knowledge, this is the first study on a combined multi-omics-based analysis to elucidate the use of GPS for NAFLD prevention.

An animal model of HFD-induced NAFLD was established to outline the etiology, histopathology, and disease progression (Recena Aydos et al., 2019). Despite known differences in the metabolic profile of human NAFLD, the HFD diet mice NAFLD model better mimics the pathological manifestations of human NAFLD compared to other dietary animal NAFLD models. This model has been widely used in preclinical drug development for NAFLD (Luo et al., 2016; Denk et al., 2019). In this study, we successfully established an NAFLD model by feeding mice with an HFD diet for 12 weeks, as evidenced by hepatic lipid deposition (increased hepatic TG and hepatic lipid droplets), and hepatic impairment (elevated serum ALT and AST levels) in HFD fed mice (Amernia et al., 2021). As a defining feature of NAFLD, hepatic steatosis develops as a result of dysregulated metabolism of *de novo* lipogenesis, fatty acid uptake, fatty acid oxidation, and TG output (Liu et al., 2015). Serum AST, ALT, and TG levels were reduced after GPS treatment compared to the HFD group. These histological observations and biochemical measurements were significantly reversed by GPS, suggesting that GPS affects lipid uptake, by attenuating hepatic steatosis, and ameliorates abnormalities in liver function metabolism in the HFD diet-induced NAFLD mice model. As the liver plays an important role in coordinating glucolipid metabolism (Ye et al., 2017), we hypothesized that GPS treatment improves lipid metabolism and reduces lipid accumulation in the liver, thus lowering lipid levels to achieve GPS-induced hepatoprotective effects.

Non-alcoholic fatty liver disease is closely related to abnormal metabolic profiles *in vivo*. To investigate the potential metabolic mechanism(s) behind GPS-mediated therapeutic effects against NAFLD, LC-MS-based serum metabolomics was performed. Herein, we observed different metabolic profiles between the four experimental groups based on the PLS-DA model. This mainly included essential amino acids *in vivo*, incompletely metabolized GPS after drug administration, and metabolites such as Cinnoline, Galabiosylceramide, and Tryptophyl-Tyrosine. Among them, Cinnolines is a heterocyclic compound with significant biological and pharmacological activity. Cinnolines also has a mildly pro-cholestatic effect and was found to be increased in the HFD feeding model. A previous study reported that disturbances in bile acid metabolism were strongly associated with NAFLD progression (Li et al., 2021). Disturbed bile acid metabolism leads to insufficient activation of the bile acid receptor FXR, which is responsible for reduced energy expenditure, increased lipogenesis, and increased bile acid synthesis that affects NAFLD progression (Clifford et al., 2021). The HFD diet reduces Galabiosylceramide levels, accompanied by activation of the sphingolipid metabolic pathway. Galabiosylceramide and FFA levels, which are part of lipid metabolism. Alterations in lipid metabolism, particularly phosphatidylcholine metabolites, are thought to be associated with liver injury, lipid peroxidation, and inflammation and therefore play a crucial role in NAFLD pathogenesis (Peng et al., 2021). Tryptophyl-Tyrosine (Trp), also known as β-indolylalanine, is an essential amino acid in humans. Gut microbiota-derived Trp metabolites have also been associated with many diseases such as inflammatory bowel disease, vascular inflammation, cardiovascular disease, liver fibrosis, obesity, and metabolic syndrome (Davidson, 2018). In addition, abnormal amino acid metabolism is common in liver injury. From metabolic pathway analysis, alanine, aspartate, and glutamate metabolism; phenylalanine, tyrosine and tryptophan biosynthesis; and phenylalanine metabolism disorders correlate with metabolic pathways in time and dose (Pannala et al., 2020). In our study, GPS supplementation reversed the abnormal levels of the metabolites induced by the HFD described above, which may explain the ameliorative effect of GPS on NAFLD development through changes related to enterohepatic circulation (bile acids), lipid metabolism (phosphatidylcholine metabolites), and tryptophan metabolism (indole derivatives).

In addition, the host gut flora was disturbed after HFD treatment (Kolodziejczyk et al., 2019). Assessment of the composition and function of the gut microbiota based on 16S rRNA sequencing revealed a decrease in the number of microbial species in the α-diversity index of the HFD diet model. Interestingly, supplementation with GPS further increased microbial richness in mice fed with the HFD diet. Cyclic enol ether terpenoids have been reported to increase the α-diversity of microbiota in HFD-fed mice (Zhang L. et al., 2021). The main phyla of gut microbiota species distribution include, Firmicutes and Bacteroidetes, followed by two minority phyla, Actinobacteriota and Proteobacteria Actinobacteria and *Aspergillus*, and the rest belong to Verrucomicrobiota and Clostridium (Delik et al., 2022), among others. In our study, Verrucomicrobiota were reduced at the gateway level in the NAFLD group. A decrease in the abundance of Verrucomicrobiota was found in mice fed a HFD for 12 weeks (Bárcena et al., 2019). Supplementation with GPS attenuated the changes in gut microbiota structure by increasing the abundance of Verrucomicrobiota. Further classification of the gut microbiota to the genus level showed that supplementation with GPS significantly increased Bacteroides and Acetobacteroides, and decreased Bifidobacterium. The ratio of thick-walled bacteria/Acetobacteria is associated with steatosis and obesity, while some other species at lower taxonomic levels are mostly associated with steatosis and obesity (Jasirwan et al., 2021). Taken together, our results suggest that the restoration of intestinal bacterial structure contributes to the beneficial effects of GPS on NAFLD by modulating the abundance of the above-mentioned flora for the purpose of treating liver injury.

Importantly, we performed a correlation analysis to clarify the interplay and regulation between metabolites and intestinal flora during NAFLD development. Importantly, in order to gain a better understanding of the mutual influence and regulation between metabolites and intestinal flora during the development of NAFLD, this study was done through correlation analysis. The correlation study between metabolites revealed that the metabolites Avenacoside B and Isoline had a significant association with almost every metabolite compared to other species. Additionally, the correlation study between intestinal flora showed that the intestinal bacteria Bacteroides and Dubosiella had strong correlations with almost every other species of intestinal bacteria. Bacteroides was positively correlated with other intestinal bacteria, while Dubosiella had a negative correlation. This suggests that despite the unclear reciprocal effects between Bacteroides and Dubosiella enterobacteria, they are in fact regulated in opposite patterns. Furthermore, the correlation analysis between metabolites and intestinal flora revealed that the metabolites Avenacoside B and Isoline were significantly correlated with almost all intestinal bacteria, and the intestinal bacterium Dubosiella likewise had a strong correlation with almost all metabolites. However, enterobacteria Bacteroides had little association with other metabolites, except for significant correlations with metabolites Avenacoside B and Isoline, likely due to its own specificity in regulating metabolites. Avenacoside B and Isoline are plant polyphenolic active components that can be obtained from malt, and have been used as antioxidants, anti-inflammatories, antibacterial, and antivirals. Meanwhile, Isoline has been used to promote cell regeneration and reduce inflammatory responses, and lower blood glucose and fat levels. Bacteroides intestinalis has been linked to high fat and protein intake, and thus plays an important role in both polysaccharides and protein metabolism. Therefore, GPS can significantly affect the levels of Avenacoside B and Isoline metabolites by modulating Bacteroides intestinal bacteria, which can have beneficial effects in lipid and hepatoprotective effects in high-fat diet mice. Results of a further correlational analysis of the mutual influence between metabolites and gut microbiota using a combined omics approach revealed that Firmicutes and Actinobacteriota bacteria were associated with the regulation of metabolites. Among them, Firmicutes are an important bacterial group directly related to the regulation of metabolites. The study found that Firmicutes had a higher expression in the control group (Figure 6A), and the quantity and diversity of the bacteria decreased significantly after high-fat diet, but it was improved after GPS treatment. This phylum contains many beneficial bacteria and some microorganisms are famous for producing butyrate, which can play a key role in the development and treatment of non-alcoholic fatty liver and type 2 diabetes through regulating metabolites. In this study, specific to metabolites and bacteria, Firmicutes gut bacteria can regulate the levels of amino acids, hormones and polysaccharides such as 2,3-Butanediolapiosylglucoside, 4-Fluoroestradiol, Glutathione thiol, etc., to inhibit the formation of free radicals and thus reduce hepatic cell damage and reduce the risk of NAFLD (Song et al., 2016). In addition, Bacteroides gut bacteria can inhibit lipid peroxidation of cells through fermentation of carbohydrates in amino acids or other classes of compounds such as Propionylcholine, Threoninyl-Lysine, Ser-Phe-OH, L-Prolinamide, D-tyrosyl-L-arginylglycyl-4-nitro-L-phenylalanyl, utilization of nitrogenous substances and biotransformation of bile acids and other classes of steroids, thus preventing the deterioration of the NAFLD process.

In conclusion, to investigate the association of GPS-mediated hepatoprotective effects with metabolism and gut flora, integrated 16S rRNA gene sequencing and LC-MS-based metabolomics were used to investigate the effects of HFD-induced NAFLD on serum metabolic phenotypes and gut microbiota. The results showed that the abundance levels of phylum and genus in the gut microbiota were significantly different. In addition, the host metabolic profile was disturbed after HFD treatment and improved after GPS treatment. Furthermore, changes in gut microbiota were significantly correlated with serum metabolite and gut flora levels, suggesting that NAFLD not only alters the gut microbiota but also affects the host metabolic phenotype, ultimately leading to dysregulation of host metabolite homeostasis. Notably, our current study has several limitations. First, although the HFD NAFLD mice model greatly mimics the pathological manifestations of human NAFLD characterized by inflammatory infiltration and hepatic steatosis, the model is not fully representative of human NAFLD. Therefore, further clinical trials of GPS are necessary if it is to be used as a potential therapy against NAFLD in the future. Second, the modulation of gut microbiota by GPS needs to be validated by expanding the sample size and germ-free mice fecal microbiota transplantation. Finally, although our results suggest that GPS therapeutic effects are associated with modulation of gut microbiota characteristics and metabolites in NAFLD mice, further studies are needed to confirm the mechanisms linking alterations in gut microbiota and host metabolome.

## 5. Conclusion

A combined metabolomics and gut microbiome analysis was used to assess the hepatoprotective effects and mechanisms of GPS on HFD-induced NAFLD in mice. We found that GPS has the potential to reduce body weight, liver index, serum AST, ALT and TG levels and improve symptoms of liver pathology. It is evident that GPS treatment helps to reduce the risk of developing NAFLD and associated with reach the desired therapeutic goals through regulating the gut microbiota Bacteroides and Firmicutes to regulate metabolites. GPS treatment can significantly affect the levels of Avenacoside B and Isoline metabolites by regulating Bacteroides gut microbiota, thus reducing the intake of fat and protein, lowering blood glucose levels, and associated with reducing blood lipid levels, thereby reducing the risk of NAFLD. In addition, Firmicutes gut microbiota can regulate the levels of various amino acids, hormones, and polysaccharides to inhibit the formation of free radicals, thus inhibiting lipid peroxidation of cells and preventing the deterioration of NAFLD process.

## Data availability statement

The data presented in the study are deposited in the National Center for Biotechnology Information repository, accession number PRJNA999357: http://www.ncbi.nlm.nih.gov/bioproject/999357.

## Ethics statement

The animal study was reviewed and approved by the All protocols of animals were approved by the Experimental Animal Ethics Committee of Changchun University of Traditional Chinese Medicine (No. 2021220).

## Author contributions

LjW and YJ completed the research and have contributed equally to this work and share first authorship. QY, CX, and JS supervised the research. LlW and YQ designed and supervised the research. All authors contributed to the article and approved the submitted version.

## Footnotes

1
https://www.metaboanalyst.ca/
