# Supplementary material for: Gentiopicroside improves high-fat diet-induced NAFLD in association with modulation of host serum metabolome and gut microbiome in mice
Source: Front Microbiol. 2023 Aug 8;14:1145430. doi: 10.3389/fmicb.2023.1145430 (PMC10443917; doi:10.3389/fmicb.2023.1145430)
Supplement: Supplementary file 2 [file Table_2.DOCX]

Supplementary Material

## Supplementary Data

Metabolites of Differences

Mod & High

Mod & Low

Con & Mod
